# Holographic Recording of Unslanted Volume Transmission Gratings in Acrylamide/Propargyl Acrylate Hydrogel Layers: Towards Nucleic Acids Biosensing

**DOI:** 10.3390/gels9090710

**Published:** 2023-09-01

**Authors:** Paola Zezza, María Isabel Lucío, Izabela Naydenova, María-José Bañuls, Ángel Maquieira

**Affiliations:** 1 Instituto Interuniversitario de Investigación de Reconocimiento Molecular y Desarrollo Tecnológico (IDM), Universitat Politécnica de Valéncia, Universitat de València, Camino de Vera s/n, 46022 Valencia, Spain; pzezza@doctor.upv.es (P.Z.); malube@upv.es (M.I.L.); amaquieira@qim.upv.es (Á.M.); 2 School of Physics and Clinical and Optometric Sciences, Technological University Dublin, City Center Campus, Central Quad, Grangegorman Lower, D07 ADY7 Dublin, Ireland; izabela.naydenova@tudublin.ie; 3 Centre for Industrial and Engineering Optics, Technological University Dublin, 13 Camden Row, D08 CKP1 Dublin, Ireland; 4 Departamento de Química, Universitat Politècnica de València, Camino de Vera s/n, 46022 Valencia, Spain

**Keywords:** holographic recording, volume transmission grating, hydrogel layer, diffraction efficiency, biosensor

## Abstract

The role of volume hydrogel holographic gratings as optical transducers in sensor devices for point-of-care applications is increasing due to their ability to be functionalized for achieving enhanced selectivity. The first step in the development of these transducers is the optimization of the holographic recording process. The optimization aims at achieving gratings with reproducible diffraction efficiency, which remains stable after reiterative washings, typically required when working with analytes of a biological nature or several step tests. The recording process of volume phase transmission gratings within Acrylamide/Propargyl Acrylate hydrogel layers reported in this work was successfully performed, and the obtained diffraction gratings were optically characterized. Unslanted volume transmission gratings were recorded in the hydrogel layers diffraction efficiencies; up to 80% were achieved. Additionally, the recorded gratings demonstrated stability in water after multiple washing steps. The hydrogels, after functionalization with oligonucleotide probes, yields a specific hybridization response, recognizing the complementary strand as demonstrated by fluorescence. Analyte-sensitive hydrogel layers with holographic structures are a promising candidate for the next generation of in vitro diagnostic tests.

## 1. Introduction

Holographic biosensors are emerging as a new technology for the development of portable analytical devices for label-free detection applications [1]. Holograms offer a direct transduction method with several advantages, such as fast response and high sensitivity. Typically, holograms are recorded in various photosensitive materials, such as silver halide films, dichromated gelatins or photopolymers [2]. Self-processing materials, such as photopolymers, are the most used in recent years, since they have excellent holographic characteristics and low cost [3]. Recently, hydrogels have attracted attention for holographic sensing applications [4]. These materials are made of three-dimensional polymeric networks of hydrophilic polymers with a high water-absorbing capacity [5]. Moreover, their composition can be fine-tuned in order to obtain appropriate chemical, mechanical and biological characteristics, enabling the incorporation of specific probes, such as oligonucleotides, proteins and others [6,7,8]. Hydrogels as support matrices for biosensing allow for the high incorporation of recognition elements in three dimensions and the provision of an aqueous and biocompatible microenvironment. For the fabrication of holographic gratings in hydrogels, it is important to obtain a transparent layer with good optical quality and high permeability. Holographic recording in light-sensitive materials is based on the process of photoinduced polymerization [9]. The fabrication method of volume holographic gratings consists of sensitizing the recording layer with a light-absorbing material and exposing it to laser light with periodically varying intensity. Normally, a mixture of monomers, a crosslinker, a free radical generator and a dye photosensitizer are required for the recording of volume transmission gratings [10]. Photopolymers are typically formed by a polymeric binder and other components that allow for the fabrication of dry layers, but the use of hydrogels as holographic matrices is strongly appealing for obtaining materials stable in aqueous environment [11,12]. On exposure to light, the photosensitizer dye reacts through electron transfer to generate free radicals so that polymerization can begin. Hence, the components of the recording material are spatially redistributed when illuminated by an optical interference pattern, resulting in a holographic volume grating. The formation of the grating involves a spatial variation in the density of the polymeric layer due to diffusion driven by the concentration gradient of monomer molecules, from non-irradiated to irradiated areas [13]. The overall refractive index is higher in the polymerized region than in the unpolymerized one due to the higher density of polymer when compared to monomer (VTG recording, Figure S1, Supporting Information). The achieved diffraction efficiency of the recorded hologram depends on many factors, such as the parameters used during recording, such as exposure time and laser intensity, as well as the chemical composition and thickness of the recording layer [14]. Holographic sensor, and biosensors in particular, are still at an early stage of development. In fact, most common applications focus on physical and chemical sensing (humidity, pH, gases and solvents), but very few are focused on biosensing [15]. The principle of the detection of holographic biosensors is based on the alteration of the diffraction efficiency when an interaction with the target biomolecule (e.g., DNA strand) occurs. Alternatively, a change in the period of the recorded grating can occur due to the material swelling or shrinkage, leading to a different position of the angle of the Bragg peak. Light-sensitive hydrogels functionalized with analyte-sensitive units represent an unexplored opportunity for the fabrication of holographic biosensors.

To date, relatively few holographic gratings have been obtained in hydrogels and used as transducers for biosensing. But this sensing technique is of great interest and potential for monitoring different targets. Notably, the transduction of the detector signal into a simple optical readout by holographic gratings can be useful for point-of-care diagnostic devices. A significant advantage of holographic gratings based on hydrogel biosensors is that they can enable the detection of analytes without labelling, thus eliminating the need for additional labels or reagents. Another beneficial aspect of hydrogel-based biosensors is that their composition can be tuned, and they can be easily functionalized with recognition molecules, including enzymes, antibodies, nucleic acids and aptamers, using conventional bio-conjugation techniques. However, there are still some challenges to overcome for the use of this methodology for applications as label-free biosensors. To achieve good analytical performance of holographic hydrogel-based biosensors, the immobilized recognition element plays a crucial role, and additional labelling and signal amplification strategies are often required. Alternatively, the performance of the hydrogel-based holographic grating can be further improved by optimizing the diffractive grating design. Another challenge is to obtain quantitative and repeatable results, due to the difficulty of controlling the quality of the gratings, especially in soft materials, such as hydrogels.

A holography-based transduction method has been successfully demonstrated for the detection of biomolecules, volatile organic compounds and metal ions [15]. For example, volume holographic gratings (VHGs) based on hydrogels as a sensing platforms have been widely employed for the measure of pH [16], humidity and temperature [17,18], metal ions [19,20], and glucose [21,22]. However, to our knowledge, the direct detection of oligonucleotides has not yet been performed by holographic sensing. This means that there are no biosensors based on this technology that can detect DNA or RNA.

This work focused on the optimization of the recording process of unslanted volume transmission gratings (VTGs) in Acrylamide/Propargyl Acrylate (AM/PA) hydrogel layers. The composition of the hydrogel had already been optimized in a previous work, in which hydrogels were surface micropatterned and utilized as biosensors [23]. By using this hydrogel composition, the bioreceptor elements (thiol-modified DNA probes) can be covalently immobilized via thiol-yne click reaction both before and after the fabrication of the volume grating. The aim of this work is to record holographic gratings in hydrogel layers with high diffraction efficiencies, for achieving an improved sensitivity of the designed holographic biosensor. In fact, phase holograms recorded in the volume of the layer offer the advantage of achieving a diffraction efficiency of nearly 100%, which is much higher than sinusoidal surface gratings with diffraction efficiencies of around 30% [16]. Herein, unslanted volume transmission gratings (VTGs) were recorded directly in (AM/PA) hydrogel layers, to the best of our knowledge, for the first time. Also, their capability to be biofunctionalized with an covalently attached oligonucleotide probe and to perform specific hybridization, thereby keeping their diffractive property, is demonstrated. Initially, (AM/PA) hydrogel layers were prepared by thermal activation. Afterwards, to carry out the recording of unslanted VTGs, the hydrogel layers were incubated in the dark with the incubation solution. This solution contains an aqueous mixture of acrylamide monomers; N, N-methylene bisacrylamide as a crosslinker; triethanolamine (TEA) as an initiator; and erythrosine B (EB) as a dye. To optimize the recording process, different concentrations of the incubation solution and recording parameters were tested. After incubation time, the hydrogels were used in holographic recording, which was observed in real time. At the end of the recording process, the gratings angular Bragg selectivity curves were characterized. Finally, the stability in water of VTGs obtained within hydrogel layers was examined in view of their potential use in biosensing.

## 2. Results and Discussions

**Figure 1:**
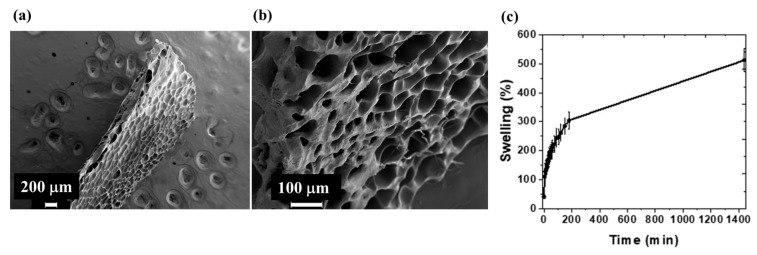
(**a**,**b**) show SEM images of the cross section of the hydrogel films at different magnifications, fully hydrated and then lyophilized, and (**c**) shows the swelling kinetic study of (AM/PA) hydrogel in PBS-T, using synthesized samples with a size of 1 cm^3^.

Thin films of hydrogels composed of AA, MBA and PA were prepared by adapting the described protocol [12,23]. AA and MBA acted as the skeletal monomer and crosslinker, respectively. PA is an additional monomer that provides the hydrogel with alkyne groups that are available to be functionalized with thiolated moieties by photo-click chemistry. We have previously optimized the relationship between monomers and crosslinkers (i.e., the crosslinking degree) needed to generate transparent and moldable hydrogels [23]. In addition, in the same study, we also fabricated hydrogels by photochemical and thermal activation. Thermal activation (i.e., using KPS as activator) yielded hydrogels with more homogenous and porous networks. Accordingly, for this work, we have chosen the previously optimized (see materials and methods section) and characterized composition and activator [23]. Once synthesized, the morphology of the lyophilized (AM/PA) hydrogel was characterized by SEM microscopy (Figure 1a). The typical porous structure of MBA-AA hydrogels of a low crosslinking degree can be observed. The ability of the hydrogel layer to swell is important to ensure that it is sufficiently permeable to facilitate the diffusion of the incubation solution. Figure 1b shows the degree of swelling of the lyophilized layers over time. These hydrogels reached approximately 500% swelling at 24 h, which is associated with the porosity of the hydrogel layer. In order to achieve a reproducible holographic recording in hydrogel layers, two different compositions of the incubation solution (Table 1) and then different recording parameters were initially tested. The hydrogel layers (AM/PA) placed on top of the microscopic slides were covered with 200 µL of incubation solution and stored inside Petri dishes in the dark at room temperature. Thus, the components of the incubation solution can penetrate the layer and thus participate in the photopolymerization. The holographic recording begins with the absorption of laser light by the sensitizing dye (erythrosine B), which is promoted to its an excited stated. It reacts then with TEA (the electron donor), producing TEA free radicals. These free radicals react with the monomer (AA) and crosslinker (MBA) to initiate free-radical polymerization. Therefore, the optical sensitivity of the hydrogel to the wavelength of the recording beam is influenced by the concentration of the components in the incubation solution. The most important aspect observed for holographic recording in hydrogels is that it is essential that the hydrogel layer reaches complete swelling in water prior to the incubation phase. In fact, no effective holographic recording was achieved for non-hydrated (AM/PA) hydrogel layers using these volumes of incubation solution. In contrast, high diffraction efficiencies were obtained for the hydrogel layers after 24 h of complete swelling in distilled water.

**Figure 2:**
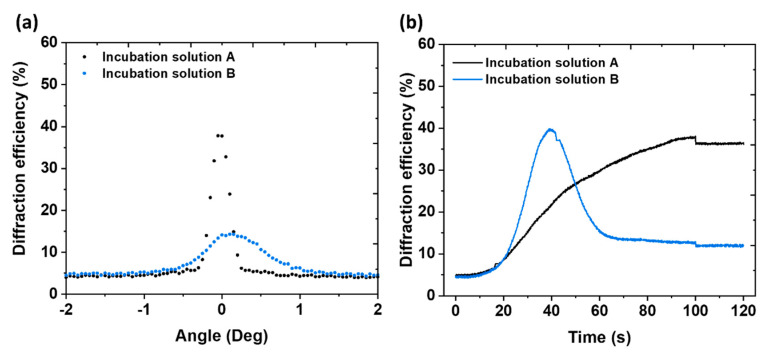
(**a**) Variation of the DE during the recording process. The real-time growth of DE in (AM/PA) hydrogel incubated with incubation solution A and B can be observed, and (**b**) variation of the DE with the incidence angle (Bragg curves) of the recorded VTGs.

Two different incubation solutions, A and B (B solution having two-fold amount of AM and MBA than A solution), were tested for the recording process. For this experiment, incubation was carried out for 1 day. The DE was monitored in real time. Better results were observed with incubation solution A. In the case of solution B, it was observed that the real-time growth curve of the diffraction efficiency initially increases and then suddenly starts decreasing (Figure 2).

**Figure 3:**
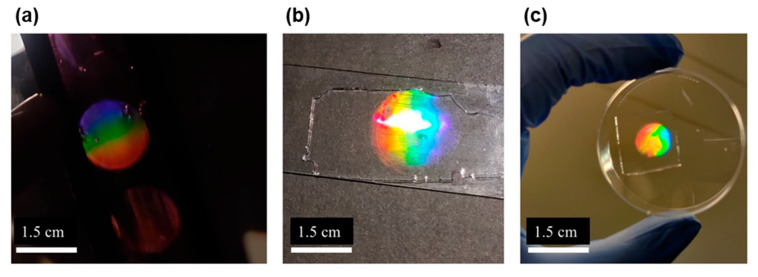
Digital photos of the transmission phase volume gratings recorded in (AM/PA) hydrogel layers: recorded spots of (**a**) 1.45 cm^2^ after recording and (**b**) 2.72 cm^2^ after first wash with distilled water; (**c**) smaller recorded spots of 0.84 cm^2^, which (image not shown), after the first washing with distilled water, reached a size of 1.56 cm^2^.

To achieve better final DE, VTG were recorded again with incubation solution B, but the laser exposure of the sample was stopped by the shutter when the diffraction efficiency started to decrease (Figure S2a). As expected, a higher DE was obtained when the exposition time was decreased (Figure S2b). The diffraction efficiency of VTG measured after the recording in the Bragg curve, where varying the incidence angle of the DE was recorded, was higher than that observed in real time during the recording process. The decrease in diffraction efficiency observed in real time (Figure 2) is most probably associated with the shrinkage of the hydrogel VTG that occurred during recording. In addition, an increased scattering of the diffraction produced by VTGs recorded with incubation solution B was observed. This behavior is due to the high concentration of monomer and crosslinker, which leads to the formation of a much harder and crosslinked VTG with a slight white color due to the increased scattering. However, it worth noting that this effect was not observed in the layers incubated with solution A. Thus, it the use of incubation solution A was decided, as maintaining the same recording parameters used with solution B resulted in holographic gratings with a higher diffraction efficiency. Furthermore, it was possible to record VTGs of different dimensions: first, spots of 1.45 cm^2^ were recorded, and later, considering their further expansion in water, smaller spots of 0.84 cm^2^ were recorded (Figure 3). This parameter is important in view of using the holograms for biosensing. For example, for the functionalization with the DNA receptor, less probe material will be required compared to the complete functionalization of the VTG area.

**Figure 4:**
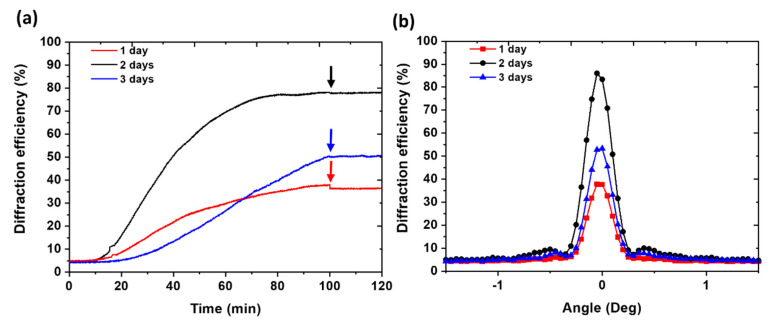
(**a**) Real-time growth curve of DE% and (**b**) the corresponding Bragg curves after one, two and three days of incubation with incubation solution A.

Further, using real-time monitoring of the recording process, different incubation times were tested with incubation solution A: one, two and three days of incubation. DE was monitored in real-time, and Bragg curves were obtained after the recording (Figure 4, Table 1). The hydrogel layers were exposed for up to 100 s during holographic recording; it was observed that, after exposure, the diffraction efficiency continues to be stable. As it can be seen from the results, the dynamic linear range increases with increasing incubation days from one to two, obtaining a DE of 80% in the latter. Interestingly, the curve of hydrogels incubated for three days showed a significant inhibition time, the hydrogel layers incubated for three days start recording the transmission gratings after 40 s of laser light exposure, whereas the hydrogels incubated for one and two days show a faster response by starting the recording process within the first 20 s of exposure. After 100 s the DE was 50% for hydrogels incubated for three days, which is lower than the DE in layers incubated for two days. The behavior of the real-time diffraction efficiency is associated with the diffusion of the incubation solution within the hydrogel, which gradually decreases over time as the hydrogel layer begins to dry out. Two days of incubation allowed for the complete absorption of the incubation solution while ensuring optimal and reproducible holographic recording with high diffraction efficiency.

**Figure 5:**
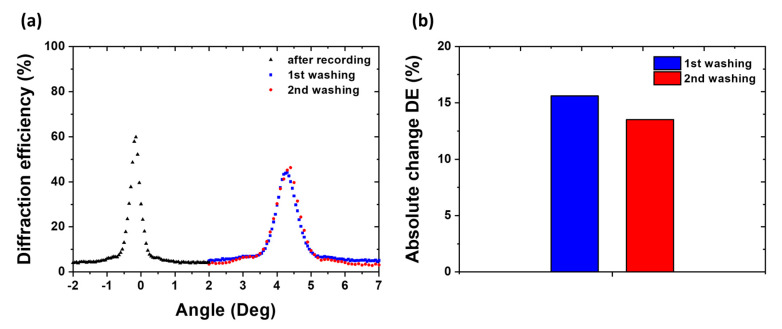
(**a**) Bragg selectivity curves of (AM/PA) hydrogel layers obtained after recording with 60% DE and after two overnight washes with approximately 44–46% DE. (**b**) Histogram of absolute DE% change after the washing steps.

To study the VTG hydrogel stability, Bragg selectivity curves were measured just after recording of hydrogels incubated for 1 day with incubation solution A and after multiple overnight washing step with distilled water (Figure 5). After the first washing step, the components that did not react during the formation of the volume transmission gratings were washed away, which is reflected in a slight change in diffraction efficiency around 10%.

The observed broadening of the Bragg selectivity curve after the washing step can be explained by the dimensional change of the layer due to the swelling of the hydrogel grating. Thus, the recorded hydrogel grating swells and expands, resulting in a slight increase in fringe spacing. The hydrogels are generally soft and elastic, and the stable and interconnected 3D crosslinked structure of the optimized hydrogel (AM/PA) allows for stable volumetric diffractive structures.

To obtain the thickness and refractive index modulation of the recorded gratings, Bragg’s angular selectivity curves were fitted using Kogelnik’s coupled-wave theory [24]. An initial simulation was performed on a VTG with 35% diffraction efficiency (Figure S3, Supporting Information). Then, the theoretical fitting was performed on the Bragg curve obtained under the optimized conditions 80% DE (Figure S4, Supporting Information). Both Bragg curves obtained from the simulation are in good agreement with the respective experimental curves. The simulation results are summarized in Table 2.

**Figure 6:**
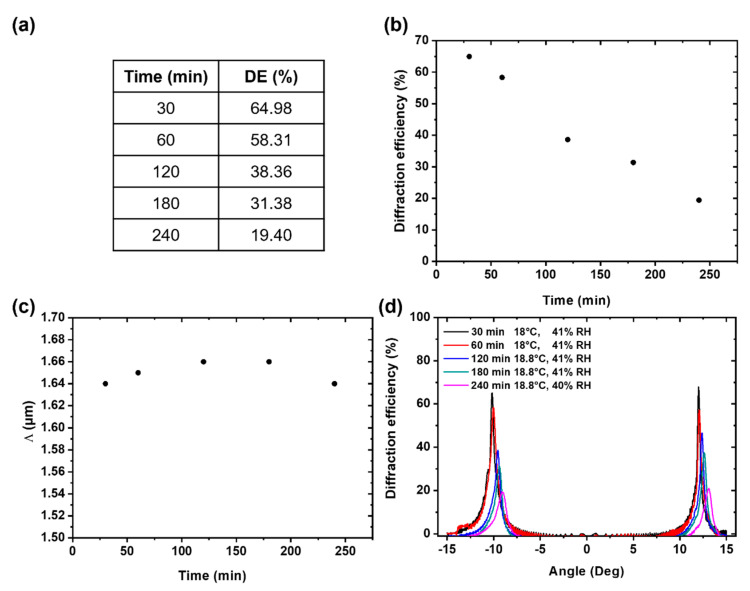
(**a**) Data of the hydration state study and (**b**) diffraction efficiency change over time. (**c**) Fringe spacing versus time and (**d**) Bragg selectivity curves of (AM/PA) hydrogel layer while drying over time.

Furthermore, the Bragg curve of the optimized volume gratings was characterized over time to test how the diffraction efficiency and/or the fringe spacing are affected when the hydrogel starts drying (Figure 6). In this experiment, the Bragg curves of the (AM/PA) hydrogel layers were obtained at controlled times and temperature and the relative humidity conditions were also monitored. The fringe spacing (Λ) was calculated with Equation (1):

(1) $$\Lambda \;=\frac{\lambda }{2\sin[\frac{\left(peak\theta 1-peak\theta 2\right)}{2}]}$$

where λ is the wavelength of the probe beam, and (peak θ_1_–peak θ_2_) is the angle calculated in the Bragg curve between the two Bragg peaks. From the results, it was possible to observe a gradual decrease in diffraction efficiency already after one hour of sample drying at RT, while the fringe spacing did not change significantly during time. It can be observed that even when the hydrogel is dry, about 30% of the initial DE% is retained. At the same time, it can also be seen that the change in the VTG period as the hydrogel dries over time is about 1%.

### Biofunctionalization of VTGs and Hybridization Assays by Fluorescence Detection

Some of the hydrogels recorded with volume gratings were subjected to the analysis of hybridization ability with complementary strands using fluorescence detection. For that, starting from a VTG with a DE of 20% in water, they were conditioned in SSC1x, and their diffraction diminished to one half. Then, VTGs conditioned in SSC1x were incubated with 5 μM solution of an oligonucleotide probe bearing a thiol group in THF/Ac-TCEP 1:1, and irradiated for 30 min, according to the protocol described for biofunctionalization [23]. After washing with SSC1x for several hours, the diffraction efficiency decreased further by 40%.

Then, the VTG hydrogel was submitted to serial incubations with increasing concentrations of Cy5-labeled oligonucleotides (0.2; 0.5; 1 and 2 μM), with the complementary sequence of the immobilized probe for 1 h. Each condition was assayed by triplicate. After each incubation, the VTG was washed with SSC1x for 1 h, and the fluorescence was recorded. The same experiment was carried out with a biofunctionalized VTG but using a non-complementary sequence for the hybridization, as a control of the specificity in the target biorecognition.

**Figure 7:**
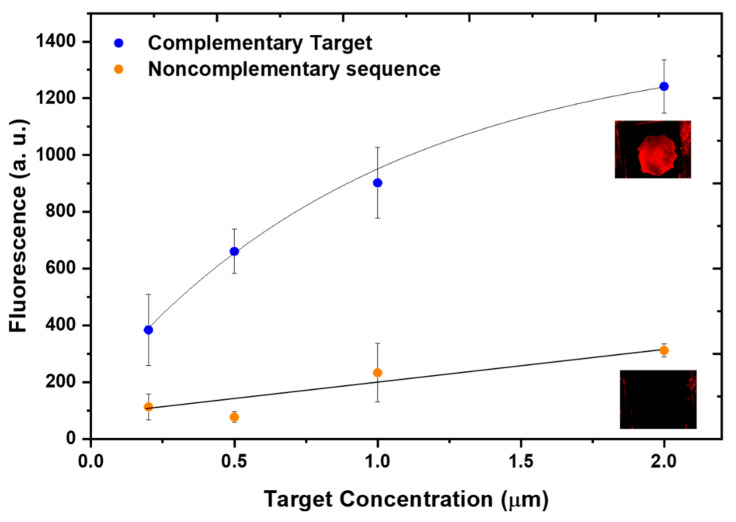
Fluorescence intensity of VTG hydrogels biofunctionalized with an oligonucleotide probe and incubated with increasing concentrations of labeled complementary target (blue) or labeled non-complementary sequence (orange) washed with SSC1x for 1h.

As it can be observed in Figure 7, the fluorescence increased with the concentration of complementary strand, while in the case of hybridization with the non-complementary target, only residual fluorescence remained inside the VTG hydrogel. Thus, it was concluded that the probe was successfully immobilized inside the VTG, keeping its bioavailability to hybridize in a specific manner. Furthermore, VTG hybridized with the complementary target keeps their diffractive capacity (Figure S5, Supporting Information).

**Figure 8:**
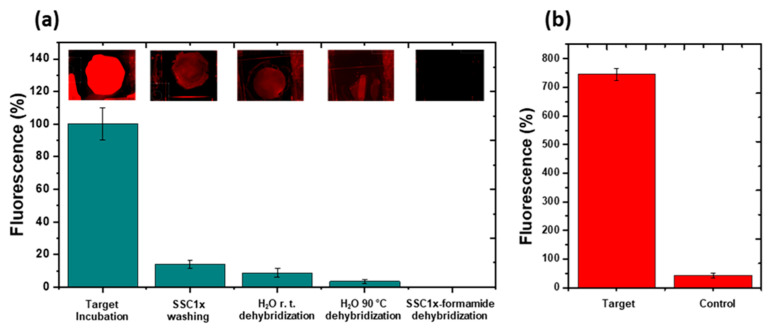
(**a**) Fluorescence (%) measured after target hybridization, washing with SSC1x, and after the several dehybridization steps carried out at different conditions. All fluorescence images were capture using the same acquisition conditions (gain and exposition time). Fluorescence was normalized to the maximum signal (target incubation). (**b**) Fluorescence of the VTG hydrogel dehybridized with SSC1x-formamide after a second cycle of hybridization with a complementary target and a non-complementary target control labeled with Cy5. Incubation was carried out for 1h in SSC1x and washing was performed for 1h in SSC1x.

To characterize the prepared hydrogels, and to demonstrate their robustness, the reusability of the biofunctionalized hydrogels was tested. For that, a biofunctionalized VTG hybridized with the complementary labeled oligonucleotide was subjected to different dehybridization conditions, and the dehybridization was monitored by fluorescence (Figure 8a). As expected, the initial fluorescence (target incubation) decreased considerably after SSC1x washing. Then, it slightly decreased after dehybridization with water washing, and even more when the temperature was increased, however, a residual fluorescence still remained inside the VTG hydrogel. This indicated that the hydrogel was not fully dehybridized. The complete dehybridization was achieved when 50% formamide in SSC1x was used, as in this case, and the fluorescence signal disappeared totally. The VTG hydrogel, fully dehybridized, was submitted to another hybridization cycle, and the corresponding control, with a non-complementary target was carried out. Figure 8b shows the fluorescence signal after washing with SSC1x. After the dehybridization, specific hybridization was again achieved, demonstrating the reusability of the bioresponsive hydrogel. Although one-shot assay is typically used in biosensing protocols, the reusability test gives a characterization of the material robustness.

## 3. Conclusions

Unslanted volume transmission gratings (VTGs) were recorded in an Acrylamide/Propargyl Acrylate hydrogel layer with good reproducibility and good optical quality. The conditions of the incubation and recording processes were successfully optimized and VTG hydrogels were optically characterized. Furthermore, the volume hydrogel gratings were found to be stable in water, maintaining their diffraction efficiency even after successive washes. The optimized holographic recording process performed in hydrogel layers can be useful for the design of potential holographic biosensors. The hydrogel VTGs can be biofunctionalized with an oligonucleotide probe, which can act as a bioresponsive material, hybridizing only with the complementary strand target and retaining their diffractive properties. The reusability of the bioresponsive hydrogel for several hybridization cycles has been also demonstrated. Thus, these three-dimensional hydrogel networks with embedded diffractive structures are promising candidates for the analysis of targets involved in diseases and health monitoring, as they are able to perform label-free detection, which can save cost and time and reduce assay complexity.

## 4. Materials and Methods

### 4.1. Materials

Acrylamide (AM) (98.5%, molecular weight (MW) = 71.08 g/mol); propargyl acrylate (PA) (98%, MW = 110.11 g/mol); N,N′-methylenebisacrylamide (MBA) (≥99.0%, MW = 154.17 g/mol); potassium persulfate (KPS) (≥99.0%, 270.32 g/mol); triethanolamine (TEA) (98%, MW = 149.19 g/mol); formamide (≥99.0%, MW = 45.04 g/mol); 2,2-Dimethoxy-2-phenylacetophenone (DMPA) (99%, MW = 256.30 g/mol); tris (2-carboxyethyl) phosphine hydrochloride (TCEP) (≥99.0%, MW = 286.65 g/mol); sodium acetate (≥99.0%, MW = 82.03 g/mol); tetrahydrofuran (THF) (99.9%, MW = 72.11 g/mol); sodium chloride (NaCl) (≥99% MW = 58.44 g/mol); sodium citrate dihydrate (≥99.0%, MW = 294.10 g/mol); ethylenediaminetetraacetic acid (EDTA) (≥99.0%, MW = 292.24 g/mol); and erythrosine B (EB) dye (≥95.0%, MW = 835.89 g/mol) were all purchased from Sigma-Aldrich (Madrid, Spain) and used without any further purification. The Ac-TCEP buffer, pH 4.5, consists of 25 mM of TCEP, 0.15 M sodium acetate, 0.1 M EDTA, and 0.1 M NaCl in DI water, and the saline-sodium citrate buffer (SSC1x, pH 7.4) consists of 0.15 M NaCl and 0.015 M sodium citrate. The oligonucleotides were supplied by Sumilab (Valencia, Spain), and the sequences used are listed in Table S1 (Supporting Information).

### 4.2. Hydrogel Layers Preparation

**Scheme 1:**
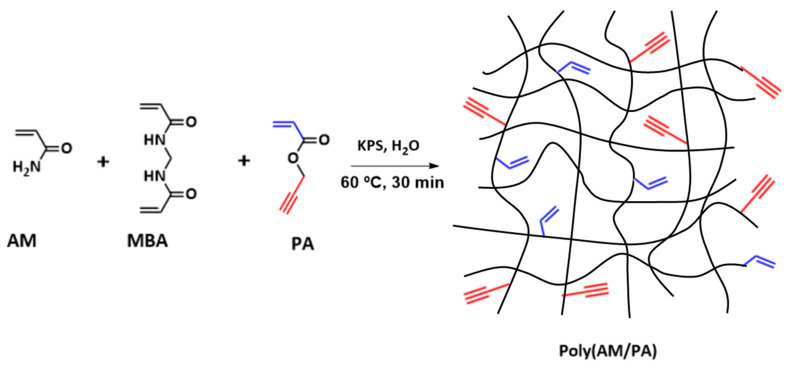
Representation of the hydrogel synthesis by free-radical polymerization (FRP). AM: Acrylamide, MBA: N,N′-methylenebis (acrylamide), PA: propargyl acrylate, KPS: potassium persulfate. The highlighted functional (acryl in blue, and propargyl in red) groups are used for the bioreceptor incorporation. Figure adapted from [23].

Hydrogel layer preparation was performed using an adapted protocol [12,23]. First, 25% (*w*/*v*) of AM, 0.05% (*w*/*v*) of MBA 75 µL of PA, and 1% (*w*/*v*) of KPS were mixed in 5 mL of distilled water. Then, (AM/PA) hydrogel layers were obtained by depositing 500 µL of the pre-polymer solution onto a well, made of a mold (5 cm × 1.4 cm × 340 µm) sticked to a levelled glass slide (7.5 cm × 2.5 cm) (Labbox Labware, S.L., SLIBG10-050, Premia de Dalt, Spain). The full well was covered with another glass slide and squeezed with two clamps. Hydrogels were synthesized by thermal activation for 30 min at 60 °C (Scheme 1). After the polymerization time, the top glass slide was removed, and hydrogels were soaked overnight in distilled water at RT. The calculated thickness of the hydrogel layer was approximately 300 ± 10 µm.

### 4.3. Morphology Characterization 

The morphological characterization of (AM/PA) hydrogel layers was performed using scanning electron microscopy (SEM, Gemini SEM 500 system, Zeiss, Oxford Instruments, Oxford, UK). First, hydrogels layers were immersed in distilled water to completely swell. Then, they were frozen at −20 °C prior to lyophilization overnight (Telstar Lyoquest freeze-drier, Azbil Telstar Technologies, S. L. U., Terrasa, Spain). The resultant dry aerogel samples were finally covered with a Au layer of about 15 nm using sputter-coating (BAL-TEC SCD 005 sputter coater, Leica microsystems, Wetzlar, Germany).

### 4.4. Swelling Behavior Studies

The swelling kinetics were obtained for the (AM/PA) hydrogel. Freeze-dried hydrogel samples of approximately 1 cm^3^ were used for this study. The lyophilized samples were immersed in PBS-T (10 mL) at RT, and their weight was recorded successively over time until a constant weight (total swelling) was reached. The degree of swelling (%) was calculated using Equation (2), where Wt is the weight of the hydrogel after being immersed in the buffer for a time ‘t’, and W_0_ is the weight of the lyophilized hydrogel before buffer immersion.

(2) $$\mathrm{Swelling}\;(\%)=\frac{Wt-Wo}{Wo}\;\cdot \;100$$

### 4.5. Incubation Step before Recording

Hydrogels layers were prepared for the holographic recording process. Two different incubation solutions were tested (A and B, Table 3). The incubation solutions contained an aqueous mixture of acrylamide; N, N-methylene bisacrylamide as a crosslinker; Triethanolamine (TEA) as an initiator; and Erythrosine B (EB) as a dye. The Erythrosine B (EB) dye was previously dissolved in distilled water at 0.11% (*w*/*v*). A volume of 200 µL of incubation solution was deposited on the already polymerized hydrogel layers. The samples then were kept inside a Petri dish in the dark at room temperature until complete absorption of the compounds within the hydrogel matrix was achieved. Different incubation times were tested: 1 day, 2 days and 3 days.

### 4.6. Holographic Recording and Probe Set-Up

**Figure 9:**
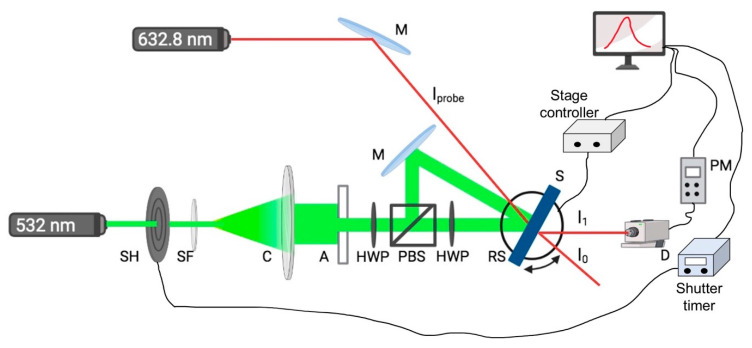
Experimental set-up for the recording of volume transmission gratings. The recording beam wavelength was 523 nm, and real-time monitoring was carried out with probe beam of 632.8 nm. M: Mirror, SH: shutter, SF: spatial filter, C: collimating lens, A: aperture, PBS: beam splitter, HWP: half wave plate; RS: rotational stage, S: sample, D: detector, PM: power meter.

The optical setup used (Figure 9) consists of a Nd:YVO_4_ laser emitting at 532 nm for the recording and a He-Ne laser emitting at 633 nm for the reading (probe laser). Two collimated beams were obtained with equal intensity by splitting the laser light from the Nd:YVO_4_ laser with a polarizing beam splitter (PBS). The intensity of the two beams was equalized with the help of a half-wave plate (HWP) positioned in front of the PBS, thus allowing for control over the state of polarization of the linearly polarized beam entering the PBS. After passing through the PBS the beam passed through a second half-waveplate. This was necessary to ensure that both recording beams have parallel polarizations for achieving maximum visibility of the interference pattern that is being recorded. Both recording beams were s-polarized. The total angle between the two recording beams of 24.6 degree was selected to create an interference pattern of a spatial frequency of 800 lines/mm (the grating period (Λ) was 1.25 µm). To ensure that the recording process was carried out properly, a He-Ne laser beam of 633 nm wavelength was used, as a probe beam, to fully characterize the holograms. The hydrogel samples were placed on a computer-controlled rotational stage (RS) (Newport ESP300). To acquire real-time diffraction efficiency growth (η) and subsequently Bragg selectivity curves, the intensity of the first-order diffracted beam (I_d_) was monitored with an optical power meter (Newport Model 840). The signal from the optical power meter was sent to an analogue-to-digital converter connected to a computer. A LabVIEW program was used to control the shutters, rotational stage, and the data acquisition. The volume transmission grating spot size was reduced form 1.45 cm^2^ to 0.84 cm^2^ using a diaphragm.

### 4.7. Holographic Recording and Characterization of Hydrogel Layers

Holographic volume gratings were recorded within hydrogel layers using transmission geometry (Figure S1a). Specifically, phase holograms were recorded through the laser-light-induced photopolymerization process, which led to the modulation of the refractive index within the hydrogel layer. Volume transmission gratings (VTGs) were recorded directly on Acrylamide/Propargyl Acrylate hydrogel layers using the unslanted configuration, in which the two recording beams have equal angles of incidence. Recording parameters, such as green laser power and exposure time, were optimized for the recording process. The development of the hydrogel holographic gratings was monitored by measuring the diffraction efficiency (DE% or η) growth curves in real time with the probe beam (red). The diffraction efficiency of the recorded VTGs was calculated as the ratio of the diffracted beam intensity (I_d_) to the incident beam intensity (I_probe_) per cent. To observe the dependence of the intensity of the diffracted light (I_d_) on the angle of incidence of the probe beam, Bragg selectivity curves of hydrogel VTGs were obtained by rotating the sample holder placed on a high accuracy rotational stage (model Newport ESP300 with angular resolution of 0.001°). After recording, hydrogels were immersed in distilled water and the diffraction efficiency was measured after several washes to verify the stability of the recorded VTGs. In addition, Kogelnik’s coupled-wave theory was used to fit the Bragg angular selectivity curves of the gratings and thus extract the thickness of the layers and the refractive index modulation created during the recording process [24].

### 4.8. Biofuncionalization of VTGs and Hybridization/Dehybridization Experiments

For future biosensing applications, the VTG layers were covalently functionalized with a thiol-modified DNA probe via the thiol-ene/thiol-yne-coupling photo-click reaction [23]. A 1 mL solution of a 1:1 THF/Ac-TCEP mixture at 5 μM of functionalization probe was prepared, and 1% (*w*/*v*) DMPA photoinitiator was added. VTG hydrogels were placed inside circular containers with 300 μL of the prepared probe solution and irradiated at 365 nm in a UV photoreactor, LightOx PhotoReact (13 mW/cm^2^ light power) (Sigma-Aldrich, Madrid, Spain) for 30 min. Then, biofunctionalized VTGs were washed overnight in SSC1x. DE was monitored before and after the biofunctionalization in SSC1x. Biofunctionalized VTGs were incubated with serial increasing concentrations of Cy5-labeled complementary or non-complementary sequence as a target (0.2; 0.5; 1 and 2 μM) for 1h and washed for 1 h with SSC1x. The fluorescence signal was monitored after every washing step using a homemade surface fluorescence reader (SFR) equipped with a CCD camera (*λ* = 647 nm, exposure time = 10 s, gain = 1). Fluorescence image data processing was performed with the GenePix Pro 4.0 software from Molecular Devices, Inc. (Sunnyvale, CA, USA). The probe and targets used are listed in Table S1 (Supporting Information). Dehybridization of VTGs hybridized with 1 μM of labeled complementary target after their washing with SSC1x was carried out by different approximations: (a) the immersion of the VTGs in 5 mL of H_2_O for 16 h at RT; (b) the immersion of the VTGs in 5 mL of H_2_O for 1 h at 90 °C; and (c) the immersion in 5 mL SSC1x, 50% formamide. Fluorescence was registered after every step (*λ* = 647 nm, exposure time = 5 s, gain = 1). A second hybridization step was carried out with the VTGs dehybridized with SSC1x, 50% formamide, with labeled complementary and not-complementary target at 5 µM, following the previously described protocol. Fluorescence was registered after washing with SSC1x (*λ* = 647 nm, exposure time = 5 s, gain = 1).

## Figures and Tables

**Table 1:** Characterization of unslanted volume grating obtained after delivering 200 µL of incubation solution A and evaluating different incubation times.

Incubation Time (Days)	Layer Thickness (µm)	Recording Beams Intensity (mW/cm^2^)	Recording Exposure Time (s)	Maximum DE Achieved (%)
1	300	7.5	100	40
2	300	7.5	100	80
3	300	7.5	100	50

**Table 2:** Results obtained by fitting Bragg selectivity curves.

	VTG Thickness (µm)	Refractive Index Modulation (RIM)
After recording	190	0.0010
First washing	60	0.0015
Second washing	62	0.0017

**Table 3:** Composition of incubation solutions.

Incubation Solution	AM (g)	MBA (g)	TEA (mL)	EB (mL)
A	1	0.2	1	4
B	2	0.4	1	4

## Data Availability

Not applicable.

## Supplementary Materials

The following supporting information can be downloaded at: https://www.mdpi.com/article/10.3390/gels9090710/s1, Figure S1: Recording and probing of a volume transmission holographic grating, and probe beam path inside the layer, *θ_B_* Bragg angle; Table S1: Sequences of DNA used; Figure S2: Real-time growth curves of diffraction efficiency, and Bragg curves of (AM/PA) hydrogel incubated with incubation solution B; Figure S3: Theoretical and experimental angular selectivity curves for VTG recorded in hydrogel layers (AM/PA); Figure S4: Theoretical and experimental angular selectivity curves for the optimised conditions for VTG recorded in hydrogel layers (AM/PA); Figure S5: Diffraction pattern projected on a white screen of biofunctionalized VTG hydrogel [24,25].
