# Supplementary material for: Holographic Recording of Unslanted Volume Transmission Gratings in Acrylamide/Propargyl Acrylate Hydrogel Layers: Towards Nucleic Acids Biosensing
Source: Gels. 2023 Sep 1;9(9):710. doi: 10.3390/gels9090710 (PMC10528564; doi:10.3390/gels9090710)
Supplement: Supplementary file 1 [file gels-09-00710-s001.zip › gels-2555868-supplementary.pdf]

Supporting Information

Holographic recording of unslanted volume transmission gratings in Acrylamide/Propargyl acrylate hydrogel layers: towards nucleic acids biosensing

Paola Zezza[1,3,4], María Isabel Lucio[1], Izabela Naydenova[3,4], María-José Bañuls*[1,2], Ángel Maquieira[1,2],
[1] Instituto Interuniversitario de Investigación de Reconocimiento Molecular y Desarrollo Tecnológico (IDM), Universitat Politécnica de Valéncia, Universitat de Valéncia, Camino de Vera s/n, 46022, Valencia, Spain; pzezza@doctor.upv.es (P.Z.); malube@upv.es (M.I.L.); amaquieira@qim.upv.es (Á.M.)
[2] Departamento de Quimica, Universitat Politécnica de Valéncia, Camino de Vera s/n, 46022, Valencia, Spain
[3] School of Physics and Clinical and Optometric Sciences, Technological University Dublin, City Center Campus, Dublin, Ireland; zabela.naydenova@tudublin.ie
[4] Centre for Industrial and Engineering Optics, Technological University Dublin, City Center Campus, Dublin, Ireland.
* Correspondence: mbpolo@upv.es

Volume transmission holographic gratings (VTG) recording

Volume phase transmission holographic gratings can be recorded after illumination of the photosensitive material with an interference pattern created by two coherent light beams as shown in Figure S1a [25]. The spatially varying light intensity leads to a spatially varying refractive index and/or thickness of the photosensitive layer. Thus, when the layer is illuminated with one of the recording beams, the light is diffracted, and the second beam is reconstructed (Figure S1b). The grating can diffract light when illuminated with a probe beam of a different wavelength l_p incident near Bragg angle. Maximum diffraction efficiency is observed when the recorded grating is illuminated at q_B (Bragg angle, Figure S1c), which is fulfilling the Bragg condition (Equation (S1)):

$$2\Lambda \sin \theta B = \lambda p, \qquad \text{(S1)}$$

Where L is the grating period, l_p is the wavelength inside the layer.
The diffraction efficiency of the grating *h* is defined as the ratio of the diffracted beam intensity I_d and the probe beam intensity I_0, (Equation (S2)):

$$\eta = Id/Io, \qquad \text{(S2)}$$

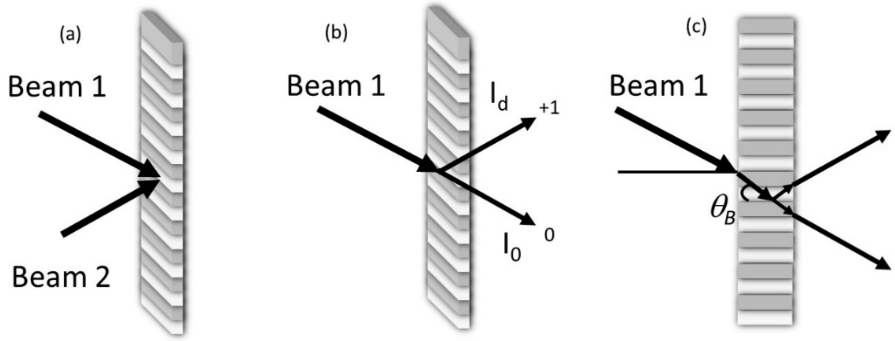

Figure S1. (a) recording and (b) probing of a volume transmission holographic grating. (c) Probe beam path inside the layer, θ_B Bragg angle.

Any deviation from Bragg angle corresponding to the specific probe wavelength will lead to a decrease of the measured diffraction efficiency. The curve presenting the dependence of the diffraction efficiency of the holographic grating on the probe beam incident angle is called angular Bragg selectivity curve. It is often used to characterise the recorded structure and any dimensional and refractive index changes occurring in response to the presence of the analyte can be observed as changes in height and/or position of the Bragg angular selectivity curve.

Table S1. Sequence of DNA used for biofunctionalization of the VTGs and biosensing assays.

**Name**	**Sequence (5′ to 3′)**	**5′ end**	**3′end**
Functionalization Probe	CCCGATTGACCAGCTAGCATT	SH	None
Complementary target	AATGCTAGCTGGTCAATCGGG	Cy5	None
Non-complementary target (negative control)	CCCGATTGACCAGCTAGCATT	Cy5	None

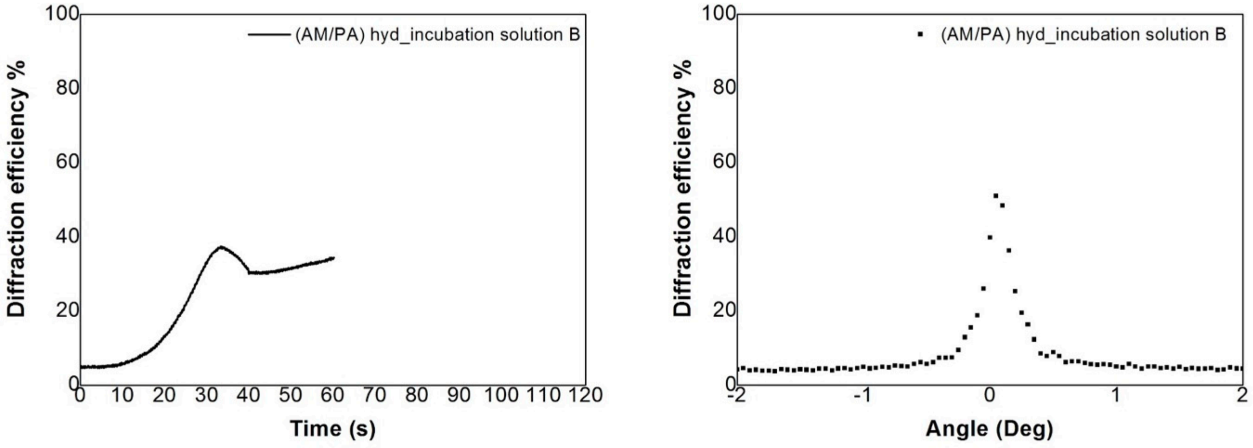

Figure S2. On the left, real-time growth curves of diffraction efficiency of (AM/PA) hydrogel incubated with incubation solution B (the laser exposure of the sample was stopped by the shutter when the diffraction efficiency started to decrease). On the right, the corresponding Bragg curves registered after recording.

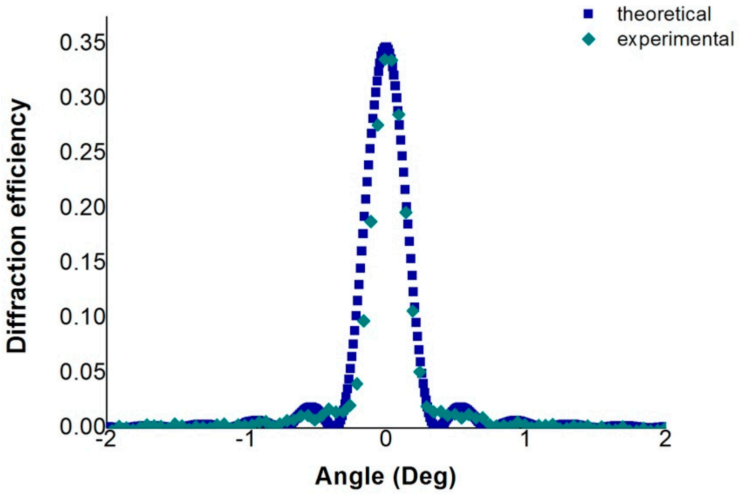

Figure S3. Theoretical and experimental angular selectivity curves for VTG recorded in hydrogel layers (AM/PA). The two curves are in good agreement. The theoretical fit is obtained according to Kogelnik's coupled wave theory for volume phase gratings [24] resulting in a hydrogel thickness of 190 μm and a refractive index modulation (RIM) of 0.000517.

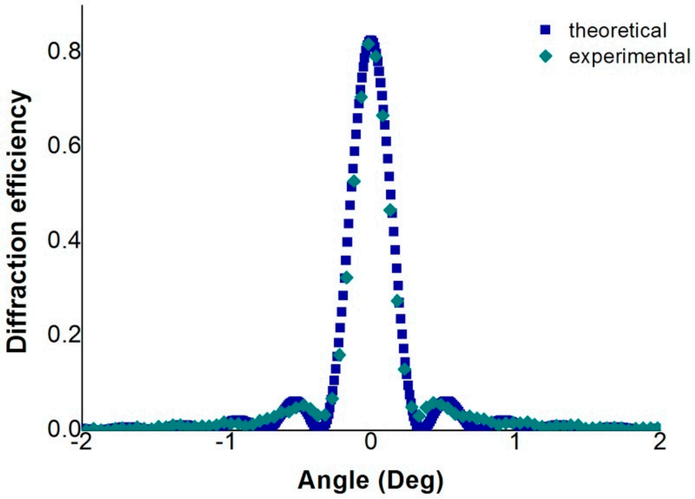

Figure S4. Theoretical and experimental angular selectivity curves for the optimised conditions for VTG recorded in hydrogel layers (AM/PA). The results show a good fit between the two curves. Fitting results showed a hydrogel thickness of 190 μm and a refractive index modulation (RIM) of 0.000963.

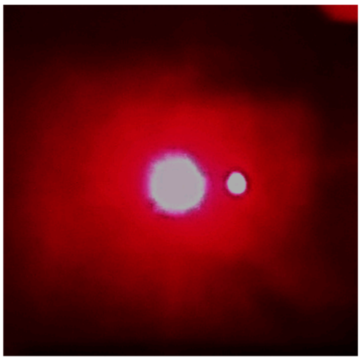

Figure S5. Diffraction pattern projected on a white screen of biofunctionalized VTG hydrogel functionalized with the thiolated probe after its hybridization with 2 μM of the complementary target and washing with SSC1x. VTG was surrounded by SSC1x and illuminated with the probe laser beam at 633 nm.

References (in manuscript text):

[24] Kogelnik, H. Coupled Wave Theory for Thick Hologram Gratings. *Bell Syst. Tech. J.* **1969**, 48, 2909–2947.

[25] Toal, V. *Introduction to Holography,* 2nd ed.; CRC Press: Boca Raton, FL, USA, 2022. https://doi.org/10.1201/9781003155416.
